# Extreme value analysis of the number of student absences in Jiangsu, China: Based on extreme value theory

**DOI:** 10.1371/journal.pone.0302360

**Published:** 2024-05-20

**Authors:** Mao Liu, Wenyi Yang, Ting Tian, Jie Yang, Zhen Ding

**Affiliations:** Jiangsu Provincial Center for Disease Control and Prevention, Nanjing, China; Australian University - Kuwait, KUWAIT

## Abstract

Attendance absences have a substantial impact on student’s future physical and mental health as well as academic progress. Numerous personal, familial, and social issues are among the causes of student absences. Any kind of absence from school should be minimized. Extremely high rates of student absences may indicate the abrupt commencement of a serious school health crisis or public health crisis, such as the spread of tuberculosis or COVID-19, which provides school health professionals with an early warning. We take the extreme values in absence data as the object and attempt to apply the extreme value theory (EVT) to describe the distribution of extreme values. This study aims to predict extreme instances of student absences. School health professionals can take preventative measures to reduce future excessive absences, according to the predicted results. Five statistical distributions were applied to individually characterize the extreme values. Our findings suggest that EVT is a useful tool for predicting extreme student absences, thereby aiding preventative measures in public health.

## Introduction

A student is generally considered absent when they fail to show up for class on time, regardless of the cause. The fact that absences might result in poorer academic performance is crucial to parents of elementary and middle school pupils, who will go out of their way to prevent absences [1]. Studies [2–8] have also shown that absences from school might lead to harmful behavioral lifestyle practices and negative health outcomes, in addition to lower grades. Students can begin experiencing absence as early as kindergarten. Research has revealed that this can lead to dropping out of school, repeating grades, or receiving poorer grades in the future. Moreover, absence can negatively impact the development of social and reading skills, putting students who are frequently absent at higher risk for exposure to unhealthy adult behaviors like smoking and drug use. Students who miss more than two weeks of classes in a given semester are at a higher risk of failing their exams when compared to their peers who consistently attend the school’s academic program. Furthermore, individuals with lower levels of education may have fewer opportunities for lucrative or socially desirable jobs, which may be associated with poorer health outcomes in the future. The study compared causes of death over the past 20 years and found that the gap in mortality rates by education level is widening. These studies attest to the importance of guarding against and avoiding student absences. There are various personal, familial, and social factors that can contribute to student absences [1]. Illness is the primary cause of student absences, and personal reasons make up the majority of these absences. During infectious disease epidemics such as influenza, tuberculosis, and COVID-19, a significant number of students are unable to attend school due to illness or risk factors [9–11]. This provides a reminder that a spike in the number of student absences could be a sign of a public health or school health crisis. Therefore, we wondered whether it was conceivable to evaluate the distribution of maximum numbers of student absences in order to anticipate such extreme levels in advance. Based on the outcomes of the predictions, we can avert potential public health or school health problems as early as possible and lessen any potential negative consequences for kids.

The extreme values are the focus of extreme value theory (EVT), which enables evaluation and risk prediction of the occurrence of extreme values. EVT has been applied to analyze the possibility that severe events may occur in a variety of domains (such as financial risk [12–15], hydrological and environmental forecasts [16–21], road traffic etc.). In contrast, there haven’t been many cases of EVT being applied to public health [22]. Our study aimed to investigate how well EVT can be applied to extreme values of student absences, find valuable tools for the analysis of extreme values of student absences, predict potential extreme values, and offer early warning recommendations for potential unexpected public health or school health events.

The remainder of the article is presented below. Section 2 includes the statistical description of the data, followed by the EVT application methods. Section 3 shows results of the extreme value analysis specifically. Finally, Sections 4 and 5 contain the discussion and conclusion, respectively.

## Related work

The related work section consists of four parts: Background of the Student Absence Monitoring Program, Development of Extreme Value Theory, Applications of Extreme Value Theory, and Applicability of Extreme Value Theory in this study. We provided a brief overview of the student monitoring program’s background, the evolution of extreme value theory, and its present application. Finally, we analyzed the applicability of extreme value theory in analyzing extreme absence values.

### Background of the student absence monitoring program

According to the Law on the Prevention of Infectious Disease, the Regulations on Emergency Measures for Public Health Emergency, the Regulations on School Health Work, and the Code of Practice for the Notification of Infectious Disease Outbreak (for trial), Jiangsu Provincial Center for Disease Control and Prevention is implementing a program to monitor absenteeism at all Jiangsu schools. The surveillance program’s objective is to gather and assess timely data on student absenteeism. Its purpose is twofold: first, to thwart any public health emergencies from developing or spreading within schools, and second, to mitigate the detrimental consequences of those emergencies. Monitoring occurs on all school days in the semester except for holidays. The manager of the monitoring program of the school reports the data of the absence monitoring through the client of the Jiangsu Student Health Monitoring System. Professionals from the Centers for Disease Control and Prevention (CDC) at all levels throughout the province review and analyze the surveillance data reported by schools daily to promptly detect potential public health emergencies.

### Development of extreme value theory

Extreme value theory was initially introduced by the German statistician Bortkiewicz [23]. He suggested that when considering a random sample that satisfies the normal distribution, its maximum value distribution corresponds to a new distribution. Prior to this, researchers primarily used the normal distribution to deal with low-probability events or extreme values. However, several studies showed that results that conformed to the normal distribution model deviated from the actual values. In 1923, R. von Mises and E. L. Dodd carried out a study of the asymptotic distribution of sample maxima for both the normal distribution and the general distribution [24]. In 1925, Tippett analyzed the maximum of a normally distributed sample and obtained a table of corresponding probability distributions [25]. Following this, Frechet proposed that samples with varying distributions could potentially have the same asymptotic distribution for their extreme values [26]. The emergence of Frechet, Weibull, and Gumbel distributions played a pivotal role in the advancement of extreme value theory [27]. Since then, an increasing number of scholars have focused on the advancement of extreme value theory. Early extreme value theory focused on the asymptotic distributions of the maximum or minimum values of identically distributed random variables. Researchers began including tails above or below a specific value in the extreme value range due to the lower utilization of the study’s data. This approach led to the emergence of peaks over threshold and multivariate extreme value distributions. Parameter estimation methods for extreme value distributions are continuously updated and refined as extreme value theory develops. Apart from the traditional maximum likelihood estimation method, available methods for parameter estimation comprise least squares, Pickands estimation, Bayesian estimation, nonlinear least squares, pot-WNLS, and more [28–31].

### Applications of extreme value theory

With the rapid development of Extreme Value Theory (EVT), it has seen an increasing application in various fields. In 1983, Vanalderwegen utilized EVT analytical methods in land planning [32], while in 1989, Papic applied EVT statistics to estimate construction machinery’s reliability characteristics and maintenance [33]. In 1994, Chryssolouris utilized extreme value theory in engineering decision making [34]. Two years later, Harris employed the statistical methods of extreme value theory in observing wind speed [35]. The study of extreme value theory initially began with asymptotic distributions of a single random variable and has gradually evolved into the study of extreme value distributions of multiple variables. Fisher conducted an applied study on the asymptotic distribution of order statistics and the joint distribution of correlation statistics [36,37]. Romano applied extreme value statistical methods to measure laminar imaging [38], while Cohen V utilized extreme value theory for assessing the risk in the commercial real estate market [39]. In China, research on extreme value theory began later compared to other countries and currently focuses on its application. Chen applied extreme value theory to the assessment of the probability of occurrence of extreme geomagnetic storms [40], and Gu applied the Bayesian extreme value distribution model to the theoretical and practical construction of the Third Yangtze River Bridge in Nanjing [41]. With the rapid development of extreme value theory and statistical modeling methods, the application areas of extreme value theory have expanded. It has now advanced from the initial probabilistic theory to the widely used mainstream statistical methods.

### Applicability of extreme value theory in this study

The wide scope of extreme value theory applications across various disciplines demonstrates the excellent versatility of the extreme value model when handling multiple datasets. Prior investigations employing EVT in epidemiological data predicted exceedingly high influenza-related annual mortality rates [22], maximal life expectancy of populations [42] and extreme incidence of influenza [43], showcasing the efficacy of EVT when analyzing public health data. In practice, the distribution of the original data does not require any special considerations with EVT, which eliminates the limitation of unknown distribution of student absence data. Moreover, our study results confirmed the EVT’s applicability, as it was more efficient in fitting extreme numbers of absent students compared to various other prevalent models for extreme value distribution.

## Material and methods

### Material

This study collected data on student absences between September 1, 2015, and June 30, 2017, for elementary, middle, and high school students in Jiangsu, Eastern China. There are approximately 7.6 million students enrolled in these schools. The time series excluded Saturdays, Sundays, and legally required holidays. We obtained data for 381 active observation days. The data were obtained from the student health monitoring system of the Jiangsu Provincial Center for Disease Control and Prevention.

### Methods

Based on the EVT, two modelling methods are applied for this study. One is the block maxima model (BMM) based on the generalized extreme value distribution (GEV), and the other is the peaks over threshold model (POT) based on the generalized Pareto distribution (GPD). BMM divides the observed data into fixed-size blocks and selects the maximum value of each block to compose the extreme value series. POT selects a sufficiently large value as the threshold and uses all data exceeding the threshold as the extreme value to obtain the extreme value series. Both models predict the extreme values that may occur at some point in the future.

#### BMM

The key of BMM fitting is choosing the appropriate block size. Data are often blocked in studies on a weekly, monthly, seasonal and annual basis. However, if blocks are too small, we will get a non-representative peak series, which will result in a time series with high variance. If blocks are too large, we may filter out some of the extreme values, resulting in a waste of data. It has been noted that the choice of block size is difficult in practical applications. Some studies have explored the use of 7, 14, 21, 30, 60, and 90 days as block sizes for division [44]. The block sizes examined in this study were 5, 10, 15, 20, 25, 30, 35, 40, 50, and 60 days. This was done with the assumption that kids attend school for 5 natural days per week at a set time.

The study found that the maximum or minimum value of a random sequence of other distributions such as normal, exponential, and uniform distributions conforms to a distribution of extreme values under limiting conditions [26], following a specific probability distribution, i.e., GEV.

The probability distribution of GEV is defined as

$$G(x;\mu ,\sigma ,\xi )=\exp\{-{\left(1+\xi \frac{x-\mu }{\sigma }\right)}^{-\frac{1}{\xi }}\},1+\xi \frac{x-\mu }{\sigma }>0$$ (1)

*μ* is the position parameter, *σ* is the scale parameter, and *ξ* is the shape parameter, *μ,ξ*∈*R,σ*>0. The shape parameter determines the type of GEV [45].

#### POT

The probability distribution of GPD is defined as

$$F(x)=1-\frac{N_{\mu }}{n}{\left(1+\xi \frac{x-\mu }{\sigma }\right)}^{-1/\xi },\;x>\mu$$ (2)

μ is the threshold, *σ* is the scale parameter, and *ξ* is the shape parameter, *μ*∈*R,σ*>0,*ξ*∈*R*.

A suitable threshold value can make the data distribution over the threshold consistent with the GPD. Therefore, the threshold should be adjusted to make it as small as possible, maximizing the use of the data without affecting the final model. There is no objective method for threshold selection for the GPD. The commonly used threshold selection methods are graphical methods such as the Mean Residual Life Plot (MRL), based on the mean exceedance function, and the Hill Plot [46–48]. For a suitable threshold, MRL should follow a linear trend about the threshold. For a practical application, a suitable threshold value can be selected based on the slope change point of the MRL. To achieve a reasonable threshold value, it is important for the graph to show a linear trend at the threshold value μ. In practice, some studies suggested using the 90% quantile as a threshold [49]. Y. Chiu used the 75%, 80%, 85%, 90%, 92.5%, 95%, 97.5%, and 99% quantiles as thresholds to select peak series in the study [44]. According to the MRL, our study explored the use of 75%, 77.5%, 80%, 82.5%, 85%, 87.5%, 90%, 92.5%, 95%, 97.5%, and 99% quantiles as thresholds.

We used maximum likelihood estimation (MLE) to estimate the parameters of BMM and POT [50]. The Quantile-Quantile plot (Q-Q Plot) was used to evaluate the fitting effect of GEV and GPD [51].

### Statistical analysis

EVT requires that the peak series satisfy the assumption of identical distribution and independence. We used the Wald-Wolfowitz (WW) test [52] to test the independence of the series and the Wilcoxon (WX) test to test the homogeneity of the series. We used the Anderson-Darling (AD) [53]test and the Kolmogorov-Smirnov (KS) test [54] to perform goodness-of-fit tests for both distributions. The Shapiro-Wilks (S-W) test [55] was used to test the normality of the data. We also investigated the goodness of fit of the log-normal (Log-N), exponential (Exp), and gamma (Gamma) distributions to the peak series, which were also widely applied in the fields of financial risk and hydrological forecasting [56].

We used the root mean squared error (RMSE) as a quantitative criterion to evaluate the fitting effect of the five distributions of GEV, GPD, Log-N, Exp, and Gamma. RMSE was used to quantify the difference between model estimates and empirical observations. We picked the distribution with the minimum RMSE to fit the extreme value series.

The statistical analyses for this study were carried out in R software. P less than 0.05 was defined as a significant difference.

## Results

### Data characteristics

A total of 381 days of student absence data were collected for this study, with a mean of 10,167 and a median of 9,628. There was a deviation between the median and the mean. The skewness indicated that the data distribution was shifted to the right and there was a fat tail, which indicated that the data were suitable for modeling and analyzing the extreme values with EVT. The S-W test also rejected the hypothesis of a normal distribution (Table 1).

**Table 1:** Statistical description of the observed data.

No. of Obs	Min	Max	Median	Mean	Std	Skew	Kurt	S-W (*P*-Value)
381	106	26198	9628	10167	4556	0.563	0.987	0.000

### Application of BMM

**Fig 1:**
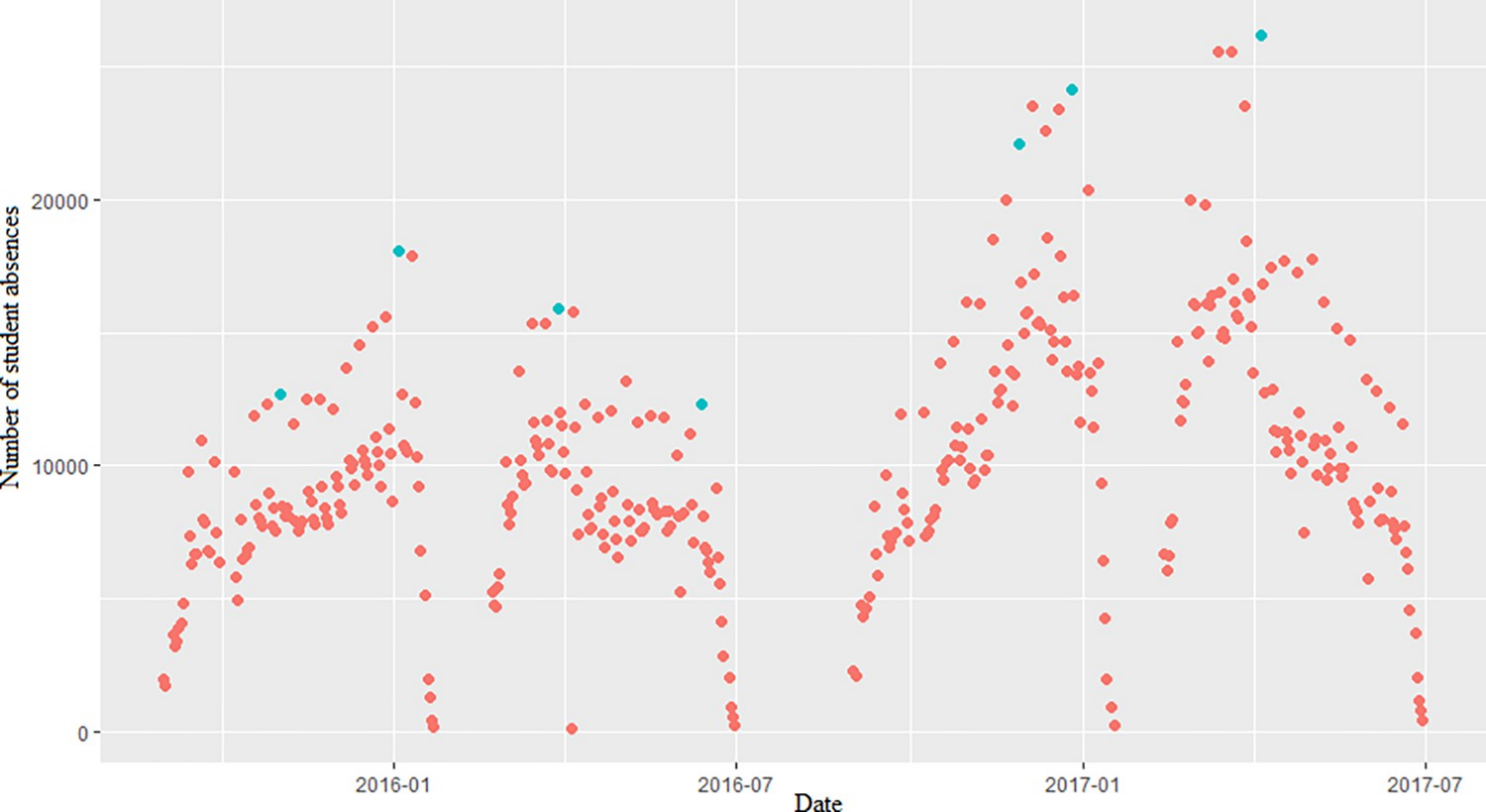
Number of student absences. Peaks of the BMM are displayed in blue.

**Fig 2:**
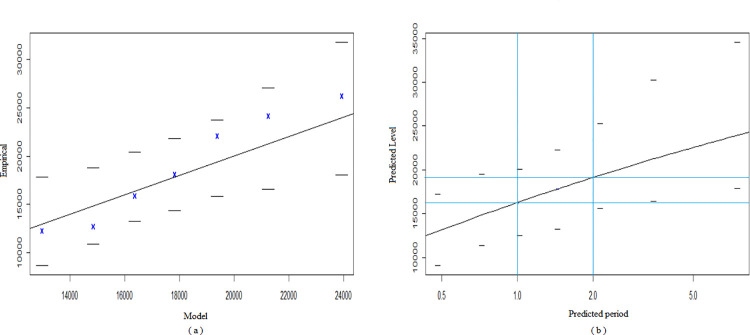
Results of the BMM. (a) Q–Q Plot for the BMM. The blue crosses depict the peaks of the fits for the BMM. (b) Predicted results for the BMM. The dashed lines represent the upper and lower limits of the confidence intervals. The blue lines indicate the maximum number of student absences that could be expected in the next 50 and 100 days.

Table 2 shows the results of the application of the BMM. We explored ten blocks of different sizes, where the peaks of 40-, 50-, and 60-day size blocks passed the WW and WX tests, and the peak sequences satisfied the requirement of being independent and homogeneous. The study separately selected the peak series of three size blocks to build the BMM, while the peak series were fitted with the Log-N, Exp, and Gamma distributions. The null hypotheses of Log-N, Exp, and Gamma distributions were rejected for the three peak series, and the null hypothesis of the GEV distribution was not rejected. We compared the RMSE of the BMM established by the three peak series, with the smallest RMSE for a block size of 50 days. We finally chose 50 days as the block size to divide the data and filtered out seven extreme values of the data. Fig 1 shows the scatter plot of the block maximum. We estimated the three parameters of the BMM, *μ* as 15,860, *σ* as 4142, and *ξ* as 0.01. The link between the model and empirical quartiles is depicted by the Q-Q Plot in Fig 2A. The relationship between the model and empirical quartiles was approximately linear, which indicated a good GEV fit. Fig 2B shows the potential extreme values predicted by the BMM for different time points in the future. The maximum number of student absences that would be expected in the next 50 days was in the range of 16,400. The maximum number of student absences that would be expected in the next 100 days was around 19,300. As time increased, the confidence interval of the model predicted values became wider and the probability of the predicted values occurring decreases.

**Table 2:** Application results of BMM.

GEV		Hypothesis (p-value)		Estimated parameters			Goodness-of-fit (p-value)		Alternative distributions (p-value)			RMSE			
block	n	WW	WX	shape	location	scale	KS	AD	Log-N	Exp	Gamma	GEV	Exp	Log-N	Gamma
5	76	0.000	0.222	-0.17	11692.90	4240.80	0.670	0.400	0	0	0	1435.78	3250.59	1111.49	1369.31
10	38	0.000	0.346	-0.19	12675.82	4214.71	0.989	0.750	0	0	0	1697.92	3509.68	1398.22	1290.02
15	25	0.000	0.713	-0.12	13155.04	4149.62	0.997	0.950	0	0	0	1487.18	3803.61	1105.35	1002.29
20	19	0.001	0.881	-0.24	13059.31	4804.34	0.832	0.700	0	0	0	2368.07	4633.36	2302.77	1814.70
25	15	0.007	0.918	-0.19	13631.87	4213.54	0.999	0.960	0	0	0	1825.06	4732.92	1206.80	1065.76
30	12	0.025	0.846	0.16	14650.00	3661.00	0.898	0.940	0	0	0	833.37	4511.60	982.70	1070.90
35	10	0.021	0.359	0.22	15510.00	4141.00	0.997	0.917	0	0	0	1437.54	5014.73	1449.57	1454.20
40	9	0.059	0.646	0.08	15750.00	4196.00	0.995	0.955	0	0	0	1549.66	5106.10	1519.70	1502.57
**50**	**7**	**0.207**	**0.575**	**0.01**	**15860.00**	**4142.00**	**0.980**	**0.966**	**0**	**0**	**0**	**1509.96**	**4969.14**	**1173.47**	**1141.02**
60	6	0.247	0.834	-0.01	15970.00	4081.00	0.963	0.685	0	0	0	1613.86	4845.75	1187.61	1184.94

### Application of POT

**Fig 3:**
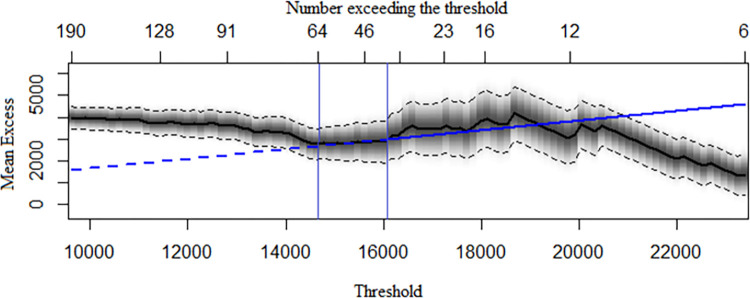
Mean Residual Life Plot (MRL) for the POT. The values between the two blue vertical lines are the initial threshold selection range recommended by MRL.

**Fig 4:**
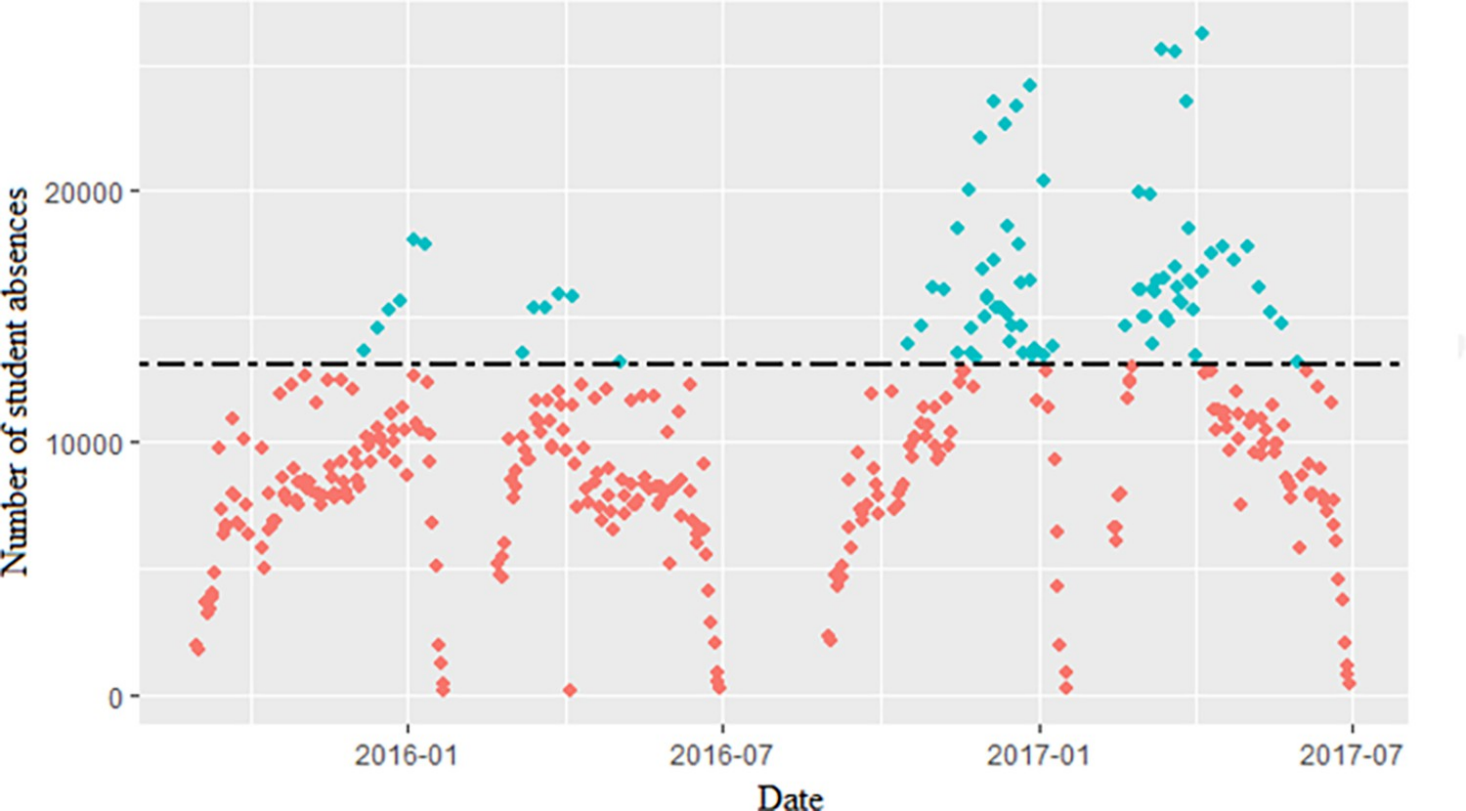
Number of student absences. Peaks exceeding the threshold are displayed in blue, while the position of the threshold is indicated by the black dashed line.

**Fig 5:**
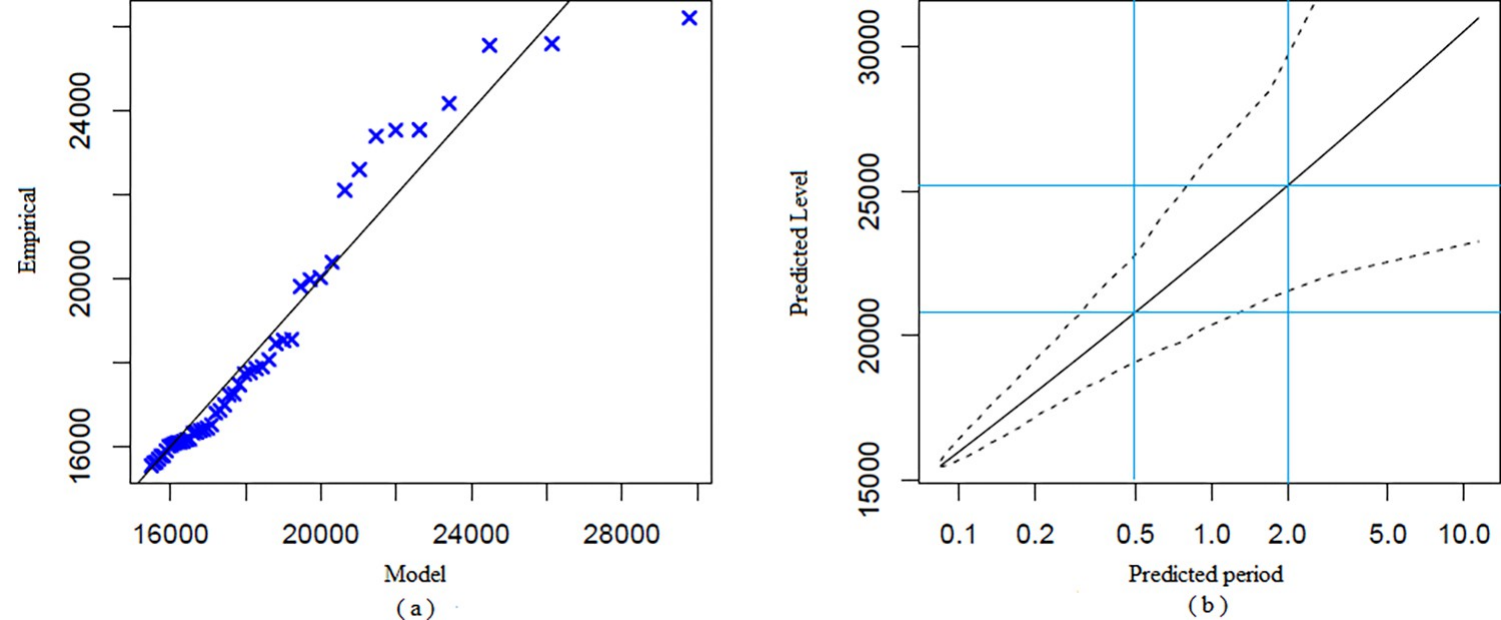
Results of the POT. (a) Q–Q plot for the POT. The blue crosses depict the peaks of the fits for the POT. (b) Predicted results for the POT. The dashed lines represent the upper and lower limits of the confidence intervals. The blue lines indicate the maximum number of student absences that could be expected over the next half and two semesters.

Table 3 displays the results of the POT application. Choosing a suitable threshold was the first step in building the POT. The optimal range for choosing the threshold is 14,500 to 16,000, according to the MRL Plot of the POT (Fig 3). We also explored the effect of fitting the GPD with eight quantiles as the threshold, with a range of 82.5% to 99% quantiles. The hypothesis that the peak sequences were independent and homogeneous was rejected by both the WW and WX tests when 82.5%, 85%, 90%, and 92.5% quantiles were used as thresholds. However, the hypothesis was not rejected when 87.5%, 95%, 97.5%, and 99% quantiles were used as thresholds. The POT had the smallest RMSE (RMSE = 287.01) when the 99% quantile (23,662) was used as the threshold, out of the four thresholds of 87.5%, 95%, 97.5%, and 99% quantiles. However, based on the MRL plots’ threshold selection recommendations (between 14,500 and 16,000) and the minimum RMSE principle, we chose the 87.5% quantile (15,464) as the threshold to extract the peak series and fitted the GPD (RMSE = 835.12). The scatter plot of the peak series over the threshold is shown in Fig 4. We got the parameter estimates of the POT with MLE, *ξ* was 0.17 and *σ* was 2358.15. The tests did not reject the null hypothesis that the peak series conformed to the GPD, but they did reject the null hypothesis that the peak series conformed to the three distributions of the Log-N, Exp, and Gamma. The Q-Q Plot in Fig 5A shows that the model overestimates the largest observed value (26,198) and that the GPD we have fitted adequately describes the distribution of extreme values. The predictions (Fig 5b) suggested that the maximum number of student absences during the next half semester (about 48 days) may exceed 20,000. Within the next two semesters, there may be a maximum of about 25,000 student absences, which is close to the maximum of the data collected in this study. The uncertainty of the prediction results increases with time. The accuracy of the predicted values is decreasing as the confidence interval for those predictions widens.

**Table 3:** Application results of POT.

GPD			Hypothesis (p-value)		Estimated parameters		Goodness-of-fit (p-value)		Alternative distributions (p-value)			RMSE			
Threshold (%)	μ	n	WW	WX	shape	scale	KS	AD	Log-N	Exp	Gamma	GPD	Exp	Log-N	Gamma
0.75	12498.0	95	0.780	0.507	-0.26	4968.91	0.736	0.613	0	0	0	678.01	3110.17	913.46	1016.82
0.775	13105.5	86	0.954	0.419	-0.20	4324.21	0.549	0.516	0	0	0	626.36	3052.25	981.81	1079.51
0.8	13570.0	76	0.699	0.309	-0.21	4346.73	0.528	0.410	0	0	0	677.02	3006.87	1051.41	1143.10
0.825	14643.0	67	0.663	0.049	0.15	2320.70	0.993	0.973	0	0	0	690.75	2986.15	1135.14	1223.46
0.85	15027.0	57	0.467	0.022	0.15	2383.86	0.982	0.969	0	0	0	747.21	3015.61	1141.13	1224.84
**0.875**	**15464.0**	**48**	**0.304**	**0.203**	**0.17**	**2358.15**	**0.960**	**0.954**	**0**	**0**	**0**	**835.12**	**3047.05**	**1125.34**	**1203.12**
0.9	16074.0	38	0.023	0.220	0.25	2240.98	0.903	0.650	0	0	0	1014.14	3113.52	1039.75	1105.81
0.925	16422.5	29	0.015	0.136	-0.11	3512.37	0.951	0.895	0	0	0	924.02	3052.03	903.61	947.50
0.95	17769.0	19	0.886	0.177	-0.35	4177.19	0.808	0.520	0	0	0	1040.71	2833.34	746.20	752.00
0.975	20204.5	10	0.515	0.878	-1.37	8232.83	0.995	0.981	0	0	0	401.74	1684.16	378.00	366.71
0.99	23662.0	4	0.420	1.000	-1.59	4020.94	1.000	0.956	0	0	0	287.01	782.14	275.46	273.82

## Discussion

In this work, we applied EVT to describe the extreme data on student absences. The study used two types of distributions that are commonly used in EVT, GEV and GPD, to fit the extreme values and create the related extreme value models. The models calculated the maximum number of student absences that may occur in the future.

The wide scope of extreme value theory applications across various disciplines demonstrates the excellent versatility of the extreme value model when handling multiple datasets. Prior investigations employing EVT in epidemiological data predicted exceedingly high influenza-related annual mortality rates [22], maximal life expectancy of populations [42] and extreme incidence of influenza [43], showcasing the efficacy of EVT when analyzing public health data. In practice, the distribution of the original data does not require any special considerations with EVT, which eliminates the limitation of unknown distribution of student absence data. Moreover, our study results confirmed the EVT’s applicability, as it was more efficient in fitting extreme numbers of absent students compared to various other prevalent models for extreme value distribution.

The key to applying EVT is the method of extracting the peak series, which also determines the type of distribution we will fit to the extreme values. In this study, we extracted the peak series by dividing the block and exceeding the threshold and we built BMM and POT, respectively. The requirements for independence and homogeneity suggest that our extraction of the peak series is not arbitrary. We selected a block size of 50 days based on the best-fit principle. According to Q-Q plots, it worked well to match GEV to extreme values of student absences. But we discovered that the large block size decreased the number of peaks we extracted. Additionally, we wasted some of the collected data as a result, and we were unable to fully utilize the data’s potential.

In contrast to BMM, POT based on GPD requires the selection of a suitable threshold. The threshold’s value is key to building the POT. A waste of data will occur if the threshold is set too big because there will be fewer data available. The bias will be greater, though, if the threshold is too small since it widens the variance between the over-threshold distribution and GPD [57]. There is no objective threshold selection method, and studies mostly used the graphical method for threshold selection [58]. In this study, we started by creating an MRL plot. After obtaining a preliminary threshold interval, we carried out the most suitable threshold exploration within it. In combination with the minimum RMSE, we used the 87.5% quantile as the threshold, which gives POT a higher usage of extreme values than BMM. In the Q-Q plot (Fig 5A), the maximum value is far from the line (*y = x*). However, we conclude that the GPD fitted by POT is successful because most of the extreme values are around the line (*y = x*).

The extreme number of absences that may occur at various points in the future was predicted by the models. Due to the two models’ different peak selection methods, we interpreted the predicted level plots of each differently. The student absence data was divided into 50-day blocks in the BMM model. These blocks were used to form a peak series by selecting the maximum values. The maximum values that may occur in the next block are the predicted values of the BMM model. In our study, the prediction time unit was defined as the block size of 50 days. When the prediction time is 1, the predicted value of the model represents the maximum number of absences that may occur in the next 50 days. When the prediction time is 2, the predicted value represents the maximum number of absences that may occur in the next 100 days. In the POT model, we divided the student absence data by semester. In China, the education department designates approximately 95 days per semester [59]. As a result, we utilized a unit of 95 days for the prediction time of the POT model in our research. In specific, a prediction time of 0.5 corresponds to the maximum number of absences that may happen in the next half semester, while a prediction time of 1 indicates the maximum number of absences during the following semester. The prediction outcomes of both models kept growing, and the 95% confidence intervals were getting broader as the prediction time points increased. This showed that, similar to other statistical prediction approaches, the forecast findings’ uncertainty increased as the prediction time increased. Despite the uncertainty in the prediction results, the predictions given by EVT still provide valuable information. EVT can be a valuable tool for exploiting the extremes of student absences in schools. In practice, school health professionals should focus more on short- or medium-term prediction levels since they are closer to the actual results. Predictions based on the extreme value model suggest that a high number of student absences are probable in the future. School health professionals can use current student absence data to compare against predicted rates. If there is a significant increase in predicted absences, they should be alert to potential short-term emergencies that may lead to further absences. It is essential to detect these events early. Many unexpected public health crises can lead to a significant boost in student absences within a brief timeframe. By swiftly recognizing the reasons behind absences, school health experts can establish preventative measures to diminish the number of student absences and decrease the risk of abrupt public health events.

Our study showed that EVT was a useful tool for processing data from school health, despite its limited application in public health. The length of the extreme series was, however, constrained by the limited duration of data collection used in this study. We assumed that the time series would remain stable throughout the investigation. The time series’ brief duration prevented us from spotting any trends. So that the GPD would fit, we decided on a fixed threshold. Plaspohl discovered that influenza had a significant impact on student absences [10]. However, we could not rule out the possibility of seasonal variations in extreme values because influenza incidence varies seasonally. Studies on the application of EVT in different industries used time-varying model parameters as well as time-varying thresholds for non-stationary time series [60,61]. In the future, as additional data is collected, we will combine the data’s properties to select a time-varying modeling method that is more appropriate. This was the first exploratory application of EVT in school health, and we did not add covariates in the study, which may affect the reliability of the prediction results. Multivariate extreme value theory encompasses models like the block component maximum model, bivariate superscalar model, and point process model [62,63], and is commonly used in the domains of hydrology, engineering, and finance. We will continue gathering and aggregating data on student absences in the future. Additionally, we have incorporated a feature that allows students to provide reasons for their absences in the absence monitoring system. This feature enables us to capture detailed accounts of the reasons behind a student’s absence. We have also initiated the collection of data on air pollution and meteorological conditions. We aim to gather comprehensive information on factors that may contribute to student absences. Our future analysis will focus on extreme cases of absences using multivariate extreme value theory, which will improve the accuracy of our predictive results.

## Conclusions

The purpose of this study was to investigate the application of EVT to build an extreme value model for the observed series of student absences. The results of the study showed that ETV was an effective tool for analyzing the extreme values in student absences. We successfully fitted the GEV and GPD to match the distribution of the extreme observations. Despite the fact that the GPD may overestimate the maximum observed value, the model still provided a valuable return level. These findings hold significant implications for school health professionals. The study further expanded the practical application of EVT in public health. While we exclusively applied EVT to student absence data in this study, we considered that EVT could also be applied to analyze other extreme observations about the school health. We will gather comprehensive information on factors that may contribute to student absences and use multivariate extreme value theory to analyze extreme cases of absences.
